# A thermodynamically consistent approach to modeling epithelial solute and water transport in the proximal convoluted tubule

**DOI:** 10.1007/s10867-026-09713-0

**Published:** 2026-06-19

**Authors:** Leyla Noroozbabaee, Jarrah M. Dowrick, Pablo J. Blanco, David P. Nickerson

**Affiliations:** 1 MERLN Institute for Technology-Inspired Regenerative Medicine, Maastricht University, Maastricht, The Netherlands; 2 Auckland Bioengineering Institute, The University of Auckland, Auckland, New Zealand; 3 National Laboratory for Scientific Computing, Petrópolis, Brazil

**Keywords:** Bond graph modeling, Kidney, Proximal convoluted tubule, Solute transport, Water transport

## Abstract

This study presents a novel approach to modeling fluid and ion transport in the proximal convoluted tubule (PCT) of the nephron using bond graphs. Bond graphs provide a robust framework for analyzing complex systems, explicitly depicting multi-domain energy exchange. Leveraging the modular nature of bond graphs, we first defined resistive modules representing membranes and capacitive modules representing solution-filled compartments, then coupled them using circuit theory. Our implementation extends beyond previous bond graph models of physiological processes by explicitly representing volumetric flow as a distinct variable within capacitive modules. In so doing, our model enables the consideration of mechanotransduction effects, where changes in fluid volume can influence membrane transporter activity, a crucial aspect of PCT function. Our bond graph model of the PCT (BG-PCT) comprises four fluid compartments bounded by five distinct membranes. The BG-PCT considers five chemical species (Na$^{+}$, K$^+$, Cl$^-$, HCO$_3^-$, and glucose) and six key membrane transporters distributed across the different membranes. Each structural subsystem comprises elementary thermodynamic processes, including dissipation, free-energy change, and power flow. This study demonstrates the advantages of bond graph modeling, particularly in its capacity to couple multiple energy domains and its modularity, which enables future extensibility. The BG-PCT provides a flexible, thermodynamically consistent platform for in silico research on epithelial transport dynamics and is available on GitHub under an open-source license to facilitate future research.

**Supplementary Information:**

The online version contains supplementary material available at 10.1007/s10867-026-09713-0.

## Introduction

Nephrons are the primary functional unit of the kidney, the organs tasked with regulating body electrolyte and fluid balance through processes including absorption and secretion [1–3]. If disturbed, the resulting imbalances of electrolytes or overall fluid volume can compromise blood pressure regulation and fundamental cellular functions [2–6]. The proximal convoluted tubule (PCT) is an essential functional segment in the nephron that plays a central role in fluid and solute transport dynamics [7, 8]. Reusable, extensible, virtual representations of the PCT can serve as powerful tools to facilitate a deeper understanding of these homeostatic mechanisms and to investigate hypotheses about pathophysiological conditions, such as hypertension, diabetes, and kidney disease [9, 10].

Several mathematical formulations have described coupled osmotic and hydraulic transport across active biological membranes. In particular, Cheng and Pinsky [11] developed a thermodynamically consistent model of fluid and osmotic pressure balance across endothelial layers, which is structurally closely related to the present formulation. At the organ scale, several models of the nephron have also been proposed using conventional kinetic modeling approaches to derive governing equations through conservation laws [9, 10, 12, 13]. However, because nephron transport inherently involves tightly coupled mechanical, chemical, and thermodynamic processes, a unified energy-consistent framework is desirable to systematically capture these multi-physics interactions.

Enter bond graphs, a domain-independent approach that exploits the universal duality between flow (e.g., current or volumetric flux) and effort (e.g., voltage or pressure) to represent energy exchange among components of a system. Using energy as the fundamental basis for modeling ensures consistent interactions between modules, enabling their independent derivation and testing before integration. Moreover, the resulting integrated system is guaranteed to behave in a thermodynamically consistent and physically plausible manner [14–17]. Hence, bond graphs offer a unified and physically consistent framework to represent the complex multi-domain interactions within a nephron, enabling modular development while ensuring the integrated system is thermodynamically consistent [17–20].

In the present study, we describe a novel bond graph model of solute and fluid transport in the PCT (BG-PCT), which provides detailed insights into the cellular and subcellular mechanisms of the transporters, solute and solvent fluxes, and energy dissipation processes. The model focuses on the membrane and solution compartment subsystems explicitly. We observed coupled dissipation within the membrane subsystems, whereas the fluid-filled compartments exhibited coupled free-energy-increasing or decreasing processes. This study showcases how modular bond graph representations of localized physical processes facilitate the analysis of complex systems in a thermodynamically consistent manner. Our model is also freely available, enabling readers to explore it by executing their own simulations and reproducibility tests [9].

## Methodology

### Thermodynamic foundations of the model

This study employs bond graphs to analyse the PCT by systematically decomposing the system into localized regions. Within each region, we examine the fundamental thermodynamic processes governing energy exchange, storage, and dissipation. These core processes are explicitly represented by specific bond graph elements, as detailed below.

Before defining the graphical bond graph elements that comprise the BG-PCT, it is important to first establish some notation. The BG-PCT model uses subscripted letters to denote compartments and membranes. For example, subscripts $\alpha$ and $\beta$ would denote distinct fluid compartments, while $\alpha \beta$ would denote the membrane separating these compartments. Unless otherwise mentioned, superscripts are used to indicate the media being transported, where *s* denotes a solute and *v* the solvent.

#### Sign convention

All flows are defined as positive in the direction indicated by the bond orientation. Negative signs in the constitutive relations arise solely from this reference orientation and ensure conservation of power within capacitive and resistive elements. This convention is applied consistently throughout all equations.

#### Bond graph elements

 

- **Bonds:** A bond (represented by a half-arrow) signifies power flow. Associated with each bond are two variables: flow and effort. These variables define the magnitude and type of power transmitted along the bond. In bond graph terminology, *effort* and *flow* are conjugate variables whose product represents power. Typical examples include voltage–current in electrical systems, pressure–volumetric flow in hydraulics, and chemical potential–molar flux in biochemical transport. This pairing ensures consistent energy accounting across physical domains and underpins the thermodynamic consistency of the model.
- **Junctions:** Junctional elements represent the topological structure of the system. These elements dictate how power flows are interconnected.
  - **0-Junctions:** Represented by **0** , 0-junctions denote parallel connections. At a 0-junction, efforts are shared (equipotential), while flows sum (analogous to Kirchhoff’s current law or mass conservation).
  - **1-Junctions:** Represented by **1** , 1-junctions represent series connections. At a 1-junction, flows are shared, while efforts sum (analogous to Kirchhoff’s voltage law).

**Table 1:**
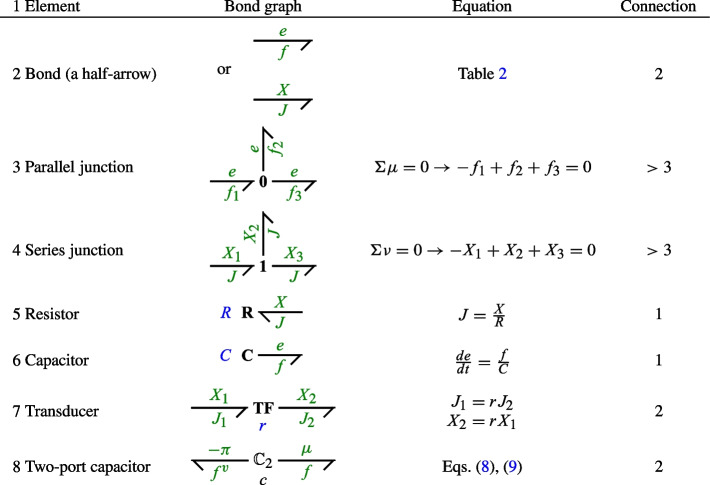
Summary of bond graph elements

**Table 2:** The following definitions are associated with the resistive module (RM) and capacitive module (CM), as depicted in Fig. 5. The CM, which represents the fluid capacitive module, is governed by the constitutive relation described by the rate of change of potential energy with respect to time, as given by (2). On the other hand, RM, representing the resistive membrane, follows the constitutive relation expressed by (1). The subscript ${\alpha \beta }$ denotes the separating membranes, which are combinations of letters indicating specific components such as the luminal cell membrane (lumen-cell membrane, MI), tight junction (ME), cell-lateral membrane (IE), interspace basement membrane (ES), or cell-basal membrane (IS). The order of the two letters indicates the positive direction of the flow

Variable	Definition	Units	Comments
*J*	Flow vector of RM$_{\alpha \beta }$	L/s, mol/s	All volumetric and solute flows
*X*	Effort vector of RM$_{\alpha \beta }$	J/L, J/mol	All pressure and electrochemical efforts
*f*	Flow vector of CM$_{\alpha }$	L/s, mol/s	All volumetric and solute flows
*e*	Potential vector of CM$_{\alpha }$	J/mol	All electrochemical and pressure potentials
*C*	Capacitive element	${\text {mol}^2/\text {J}}$	See expression (2)
*R*	Resistive element	${\text {mol}^2/\text {J}}$	See expression (1)
CM$_{\alpha }$	Solution capacitive module	–	The BG-PCT includes four CMs
RM$_{\alpha \beta }$	Membrane resistive module	–	The BG-PCT includes five RMs
$\mu _{\alpha }^{s}$	Chemical potential	J/mol	
$\overline{\mu }_{\alpha \beta }^{s}$	Electro-chemical potential	J/mol	See expression (10)
$\psi _{\alpha }$	Electrical potential	mV	See expression (20)
$p_{\alpha }$	Hydrostatic pressure	${\text {J}/\text {m}^3}$, J/L	See expression (19)
$\pi _{\alpha }$	Osmotic pressure	${\text {J}/\text {m}^3}$, J/L	See expression (11)
$J_{\alpha \beta }^{s}$	Solute flux (in RM)	mol/s	See expression (41)
$J^v_{\alpha \beta }$	Volumetric flux (in RM)	L/s, ${\text {m}^3/\text {s}}$	See expression (36)
$f_{\alpha }^{s}$	Solute flux	mol/s	
$f^v_{\alpha }$	Volumetric flux (in CM)	L/s, ${\text {m}^3/\text {s}}$	
$c_{\alpha }^{s}$	Concentration of solute *s*	mol/L	
$n_{\alpha }^{s}$	Number of moles of solute *s*	mol	See expression (49)
$V_{\alpha }$	Volume of compartment $\alpha$	${\text {m}^3}$	See expression (48)
$\overline{c}_{\alpha \beta }^{s}$	Logarithmic mean concentration	mol/L	

Generalized conduction can be represented using a lumped resistor-capacitor bond graph model, where resistors are connected in series (via 1-junctions) and capacitors are connected in parallel (via 0-junctions).
- **Resistors:** Resistance elements, denoted by **R** , are essential components in bond graph models for representing energy dissipation. Incorporating resistors enables the calculation of system efficiency and the identification of energy loss locations. The constitutive relation for membrane transport is given by (1):
  1 $$\begin{aligned} R = \frac{X}{J}, \end{aligned}$$
  where *X* is a vector of the efforts (or potential differences) across the membrane, and *J* is a vector of the flows through the membrane. Both linear and non-linear resistors are employed in the model. The specific notations used for these elements in the bond graphs are detailed in Table 1.
- **Capacitors:** Generalized capacitors (one-port capacitors), denoted by **C** , represent potential energy storage. Flow variables are represented by *f*, while effort (or potential) variables are represented by *e*. The constitutive relation for a fluid compartment, expressed in terms of the rate of change of potential energy, is as follows:
  2 $$\begin{aligned} \frac{de}{dt} = -\frac{f}{C}. \end{aligned}$$
- **Transducers:** Represented by **TF** , transducers model the conversion and transmission of power between distinct processes. In this work, we employ transformer-type transducers, which establish a proportional relationship between either efforts or flows. As two-port elements, these transducers modify the power type, achieving conversion and transfer without introducing dissipation or storage. The transformation ratio, denoted by *r*, varies depending on whether the transducer is associated with a capacitive or resistive module.
- **Capacitive module transducers:** For a capacitive module, the primary side’s independent effort variable ($e_1$) determines the secondary side’s effort ($e_2$) according to the transformation ratio (*r*). The transduced secondary power is given by the product of the secondary side’s effort and flow ($e_2f_2$). The primary side’s flow variable ($f_1$) is dependent on the secondary side’s flow ($f_2$) according to the transformation ratio ($f_1 = r f_2$). Power conservation is described by:
  3 $$\begin{aligned} e_{2} f_{2}&= r e_{1} f_{2} = e_{1} f_{1}, \end{aligned}$$
  4 $$\begin{aligned} r&= z^{s} F, \end{aligned}$$
  where $z^{s}$ represents the ionic charge of chemical species *s* and *F* denotes Faraday’s constant.
- **Resistive module transducers:** The same principles apply to resistive modules, with power conservation described by the following:
  5 $$\begin{aligned} X_{2} J_{2}&= r X_{1} J_{2} = X_{1} J_{1}, \end{aligned}$$
  6 $$\begin{aligned} r&= (1 - \sigma ^{s}_{\alpha \beta })\, \overline{c}^{s}_{\alpha \beta }. \end{aligned}$$
  where $\sigma ^s_{\alpha \beta }$ is the reflection coefficient, and $\overline{c}^s_{\alpha \beta }$ is the logarithmic mean of concentrations on both sides of the membrane. The reflection coefficient satisfies $0 \le \sigma ^{s}_{\alpha \beta } \le 1$, where $\sigma =0$ denotes complete passage (no reflection) and $\sigma =1$ denotes total reflection (no passage).
  7 $$\begin{aligned} \overline{c}^s_{\alpha \beta } = \frac{c^s_{\alpha }- c^s_{\beta }}{\ln {(c^s_{\alpha }/ c^s_{\beta })}}. \end{aligned}$$
- According to [21], in fluid compartments, a reversible two-port paired capacitive element produces a set of absolute potentials that are affected by each solute $f^{s}_{\alpha }$ and volumetric flows $f^{v}_{\alpha }$ [21]. The two-port paired element, denoted by $\mathbb {C}_{2}$ (Table 1), serves as a chemical capacitor for permeable solutes, with one port designated for solute flow and the other for volumetric flow, as concentration ($c^{s}_\alpha$) is a two-parameter function of solute and solvent. The relationship between solute flow $f^{s}_{\alpha }$, volumetric flow $f^{v}_{\alpha }$, and concentration change can be expressed as follows:
  8 $$\begin{aligned} \frac{d\pi ^{s}_{\alpha }}{dt}&= \frac{f^{v}_{\alpha } \pi ^{s}_{\alpha }}{V_\alpha } - \frac{f^{s}_{\alpha } R T}{V_\alpha },\end{aligned}$$
  9 $$\begin{aligned} \frac{d\mu ^{s}_{\alpha }}{dt}&= \frac{f^{v}_{\alpha } R T}{V_\alpha } - \frac{f^{s}_{\alpha } R T}{n^{s}_{\alpha }}, \end{aligned}$$
  10 $$\begin{aligned} \overline{\mu }^{s}_{\alpha }&= \mu ^{s}_{\alpha } + \psi _\alpha z^{s} F\end{aligned}$$
  11 $$\begin{aligned} \pi ^{s}_{\alpha }&= c^{s}_{\alpha } R T, \end{aligned}$$
  where the potentials are expressed by the osmotic pressure $\pi _{\alpha }^s$ and the chemical potential $\mu ^{s}_{\alpha }$. Here $\overline{\mu }^{s}_{\alpha }$ is defined as the electrochemical potential, as it includes both chemical potential ($\mu$) and electrical potential ($\psi$) due to the ionic charges. Here, $V_\alpha$ denotes the volume of compartment $\alpha$, $\pi _\alpha ^s$ the osmotic pressure contribution of solute *s*, *R* the universal gas constant, and *T* the absolute temperature. Equations (8), (9) are derived from the following mass conservation equation
  12 $$\begin{aligned} -\frac{d c^{s}_{\alpha }}{dt} = \frac{c^{s}_{\alpha } f^{v}_{\alpha }}{V_\alpha } - \frac{f^{s}_{\alpha }}{V_\alpha }, \end{aligned}$$
  by separately considering the osmotic and chemical potentials.

**Fig. 1:**
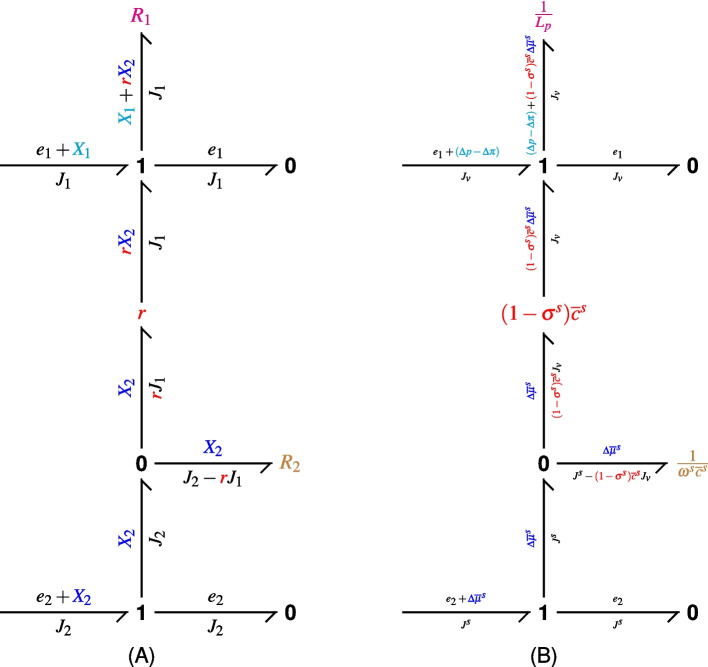
Basic coupling resistive module. **A** Bond graph representation of a simplified two-port resistive module, illustrating power coupling. This example, based on established network thermodynamics principles [21, 23, 24], comprises two resistors: $R_1$ in series and $R_2$ in parallel with a transformer-type transducer. For clarity, chemical transporters are omitted here; their integration is detailed in Fig. 4. **B** The corresponding Kedem-Katchalsky coupled transport equations derived from the resistive module in **A**. The highlighted $\textbf{0}$-junctions indicate connection points for integrating this module with other subsystems, such as solution compartments

### Modular elements of epithelial transportation

In the current BG-PCT model, we introduce resistive and capacitive modules to represent epithelial transport processes, each constructed from various bond graph elements (Fig. 1). Solute and solvent movement is driven by the following potential differences generated between solution compartments on either side of a membrane [22]:

13 $$\begin{aligned} \Delta p_{\alpha \beta }&= p_{\alpha } - p_{\beta }, \end{aligned}$$

14 $$\begin{aligned} \Delta \mu ^{s}_{\alpha \beta }&=\mu ^{s}_{\alpha }-\mu ^{s}_{\beta },\end{aligned}$$

15 $$\begin{aligned} \Delta \pi _{\alpha \beta }&=\pi _{\alpha }-\pi _{\beta }, \end{aligned}$$

where *p* represents hydrostatic pressure, $\mu ^s$ chemical potential of solute *s*, and $\pi$ the osmotic pressure. The osmotic pressure in fluid compartment $\alpha$ is defined as

16 $$\begin{aligned} \pi _{\alpha }= \sum _{s} c^{s}_{\alpha } R T. \end{aligned}$$

where *R* is the universal gas constant and *T* is absolute temperature (Table S1; Online Resource 1).

#### Resistive modules

Resistive modules simulate energy dissipation during membrane transport processes. Each resistive module consists of a hydraulic resistor representing the dissipative process of fluid flow driven by hydrostatic pressure differences, as well as solute and chemical resistors representing the dissipation associated with solute transport and chemical reactions, respectively. The number of solute resistors corresponds to the number of solutes in the epithelial system, while the number of chemical resistors corresponds to the number of membrane-bound transporters. In addition, transducers are used to couple different types of flow (e.g., volumetric to solute), ensuring physically consistent energy exchange as detailed in (5).

Figure 1 presents the structure of these resistive modules. Panel A showcases a generic two-port power coupling system, and panel B translates this abstraction into a water-solute coupled transport system (indices $\alpha \beta$ omitted for simplicity), where volumetric flow is denoted by $J^{v}$ and solute flow by $J^{s}$. The potentials driving membrane transport processes defined above are resisted by hydraulic conductivity $L^v_{\alpha \beta }$ (inverse of $R_1$) and solute permeability $\omega ^s_{\alpha \beta }$ scaled by the mean solute concentration (inverse of $R_2$). The coupling coefficient between solute and solvent domains *r* is defined in (6).

The driving force $\Delta p_{\alpha \beta } - \Delta \pi _{\alpha \beta }$ is represented by $X_1$, while $X_2$ corresponds to $\Delta \mu ^s_{\alpha _\beta }$. Flows $J^v$ and $J^s$ correspond to flows $J_1$ and $J_2$. With these instantiations, we obtain the Kedem-Katchalsky coupled transport equations from the bond graph shown in Fig. 1

17 $$\begin{aligned} J_{1}&= \frac{1}{R_1}( X_{1} + r X_{2})  &   \Rightarrow&J^v_{\alpha \beta }&= L^v_{\alpha \beta }[\Delta p_{\alpha \beta } - \Delta \pi _{\alpha \beta } + \sum _s(1 -\sigma ^{s}_{\alpha \beta }) \overline{c}^{s}_{\alpha \beta }\,\Delta \overline{\mu }^{s}_{\alpha \beta })], \end{aligned}$$

18 $$\begin{aligned} J_{2}&= r J_{1} + \frac{1}{R_2}X_{2}  &   \Rightarrow&J^{s}_{\alpha \beta }&= (1-\sigma ^{s}_{\alpha \beta }) \overline{c}^{s}_{\alpha \beta } J^v_{\alpha \beta } + \omega ^{s}_{\alpha \beta }\overline{c}^{s}_{\alpha \beta }\Delta \overline{\mu }^{s}_{\alpha \beta }. \end{aligned}$$

#### Capacitive module

The BG-PCT model contains two types of capacitive modules: the half-cell-type and the whole-cell-type [21, 22]. The half-cell-type capacitive module generates a set of absolute potentials, while the whole-cell-type capacitive module generates potential differences between two solution compartments [21]. These hydrostatic and electrochemical potential differences serve as the driving forces in the system. Here, we denote the generic compartment by $\alpha$.

**Fig. 2:**
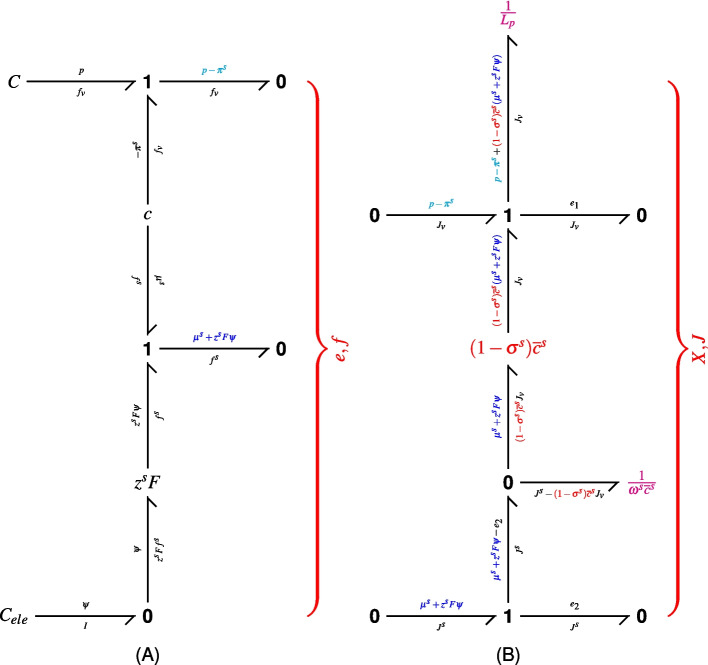
Bond graph description of the transport of water and solutes across a membrane. **A** Solution compartment modeled as a half-cell-type capacitive module. The power supply in the system is modeled using a combination of a two-port capacitor, two one-port capacitors, and a transducer. Changes in volume, the electrochemical potential of solutes, osmotic pressure, and hydrostatic pressure occur in the solution compartments. **B** Resistive module representing coupled dissipative processes in the cell membrane. This module demonstrates the dissipation of hydrostatic and electrochemical powers and the couplings between solute and volumetric flows

The half-cell-type capacitive module comprises a two-port capacitor and two one-port capacitors (each defined in Table 1). The one-port capacitive elements modulate the absolute potential in response to changes in flow. In Fig. 2A, the volume capacitor $C_{\alpha }$ and electric capacitor $C_{\alpha , ele}$ are modeled as one-port half-cell-type C-elements, according to the following equations:

19 $$\begin{aligned} \frac{dp_{\alpha }}{dt}&= -\frac{1}{C_{\alpha }} f^v_{\alpha },\end{aligned}$$

20 $$\begin{aligned} \frac{d\psi _{\alpha }}{dt}&= -\frac{1}{C_{\alpha , ele}}I_{\alpha }. \end{aligned}$$

Here, the hydrostatic pressure $p_{\alpha }$ is modulated by the volumetric flow $f^v_{\alpha }$ from the solution compartment, and the absolute electrical potential $\psi _{\alpha }$ is modulated by the current $I_{\alpha }$. A transformer-type transducer with a transfer ratio of $z^{s} F$ converts the ionic flow of $f^{s}_{\alpha }$ into the electric current given by (21), and the electric potential to chemical potential given by (22)

21 $$\begin{aligned} I_{\alpha }&=F\sum _{s}\,z^sf^{s}_{\alpha }, \end{aligned}$$

22 $$\begin{aligned} \widetilde{\mu ^{s}_{\alpha }}&= z^{s} F \psi _{\alpha }. \end{aligned}$$

Here, we also define the following quantities:

23 $$\begin{aligned} \mu ^{s}_{\alpha }&= R T \ln {c_{\alpha }^{s}},\end{aligned}$$

24 $$\begin{aligned} \overline{\mu }^{s}_{\alpha }&=\mu ^{s}_{\alpha }+ \widetilde{\mu ^{s}_{\alpha }} . \end{aligned}$$

### Transporter fluxes

The BG-PCT model contains three categories of secondary active transporters: simple co-transporters, simple exchangers, and complex exchangers. Regardless of category, these transporters are modeled using linear non-equilibrium thermodynamics, where solute permeation rates are proportional to the electrochemical potential gradients and the solute permeability coefficients. The BG-PCT model also contains one primary active transporter, Na$^+$/K$^+$-ATPase, which exchanges three cytosolic Na$^+$ ions for two peritubular K$^+$ ions.

To maintain focus on the biological relevance and physiological behavior represented by the BG-PCT model, the detailed mathematical formulations and equations governing transporter fluxes, including stoichiometries and coupling coefficients, are provided in Online Resource 1.

### Membrane epithelial transport

**Fig. 3:**
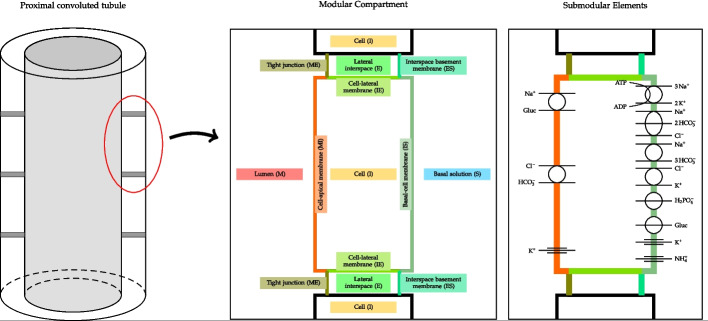
Structure of the modular and submodular elements of the proximal convoluted bond graph (BG-PCT) model. Schematic representation of the **A** proximal convoluted tubule (PCT) epithelium, illustrating the **B** compartments, solutions, and **C** transporters considered in the BG-PCT model

In the context of this study, each compartment is characterized by the concentration of different species $c_{\alpha }^{s}$. These concentrations provide information about the distribution of different ions or molecules within each compartment of the PCT system (Fig. 3). Solute and volumetric fluxes across the membranes are represented by $J^s_{\alpha \beta }$ and $J^v_{\alpha \beta }$, respectively, with $A_{\alpha \beta }$ representing the membrane surface area, $V_\alpha$ the volume, and $\psi _{\alpha \beta }$ the trans-epithelial potential difference. Scaling factors for permeability coefficients, adapted from Weinstein [25], account for effective membrane areas.

**Fig. 4:**
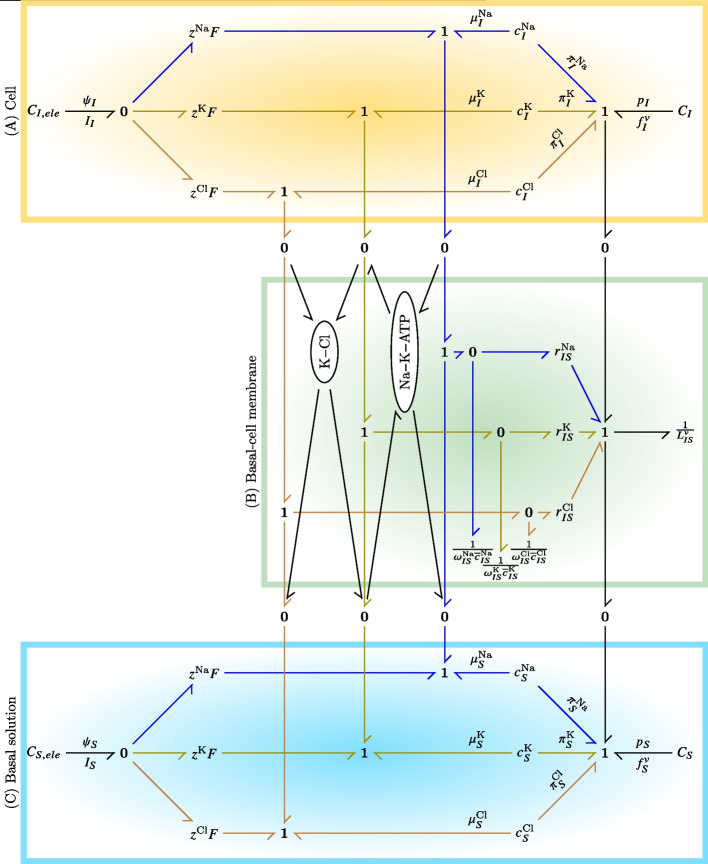
Schematic representation of the basal-cell membrane. **A** and **C** illustrate solutions in the cell and peritubular compartments, respectively. **B** shows the basal-cell membrane positioned between these two solution compartments. Only the Na$^+$/K$^+$-ATPase pump and K$^+$/Cl$^-$ co-transporter, with Na$^+$, K$^+$, and Cl$^-$ ions and water permeabilities are included to minimize visual clutter

Figure 4, a simplified bond graph representation of the basal-cell (IS) membrane and associated compartments, is included to illustrate the coupling between resistive and capacitive modules and transporters. In this example, transportation with Na$^+$/K$^+$-ATPase pump and the K$^+$/Cl$^-$ co-transporter, along with permeabilities for Na$^+$, K$^+$, and Cl$^-$ ions and water, is included. The figure effectively shows the changes in volume, electrochemical potential, osmotic pressure, and hydrostatic pressure in the solution compartments. The resistive module illustrates the dissipation of hydrostatic and ionic powers and exhibits coupling between the ionic flows and the volumetric flow. Figure 4 also highlights the interactions between Na$^+$ and K$^+$ flows in the case of the Na$^+$/K$^+$-ATPase pump and the interaction between K$^+$ and Cl$^-$ in the case of the K$^+$/Cl$^-$ co-transporter.

**Fig. 5:**
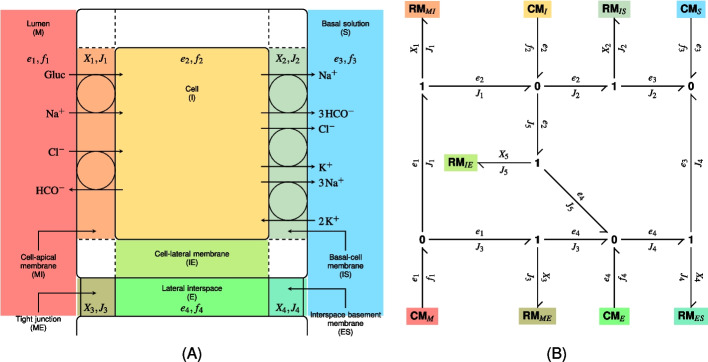
Modular representation of epithelial transport using a bond graph approach. **A** Schematic of the proximal convoluted tubule (PCT) epithelium, highlighting its cellular structure and intercellular space. The epithelium lining the lumen contains various membrane transporters, which facilitate transport processes. **B** Bond graph model of the epithelial transport system. Membranes are represented by resistive modules (RM), while intracellular, extracellular, and solution compartments are represented by capacitive modules (CM). The series connection of resistors and parallel connection of capacitors illustrate the general conduction pathways within the system. Five membranes separate these compartments: the luminal cell membrane (MI), tight junction (ME), cell-lateral membrane (IE), interspace basement membrane (ES), and basal-cell membrane (IS)

**Fig. 6:**
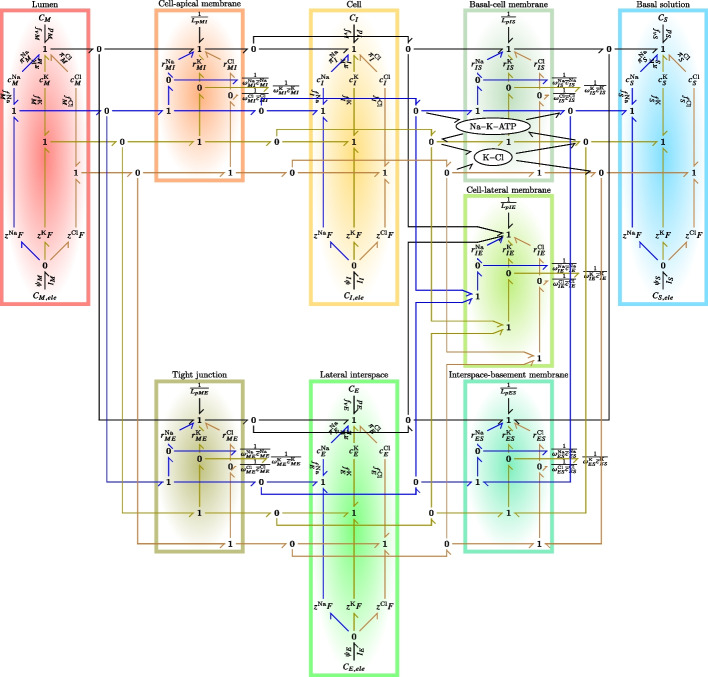
The current BG-PCT model comprises cellular and lateral intercellular compartments within the luminal and basal solutions. We consider five ion species: Na$^+$, K$^+$, Cl$^-$, HCO$_3^-$, and *Glucose*, located in four different fluid compartments (CMs) encompassing the lumen (M), lateral interspace (E), cell (I), and basal solution (S). Five membranes separate these compartments: the luminal cell membrane (MI), tight junction (ME), cell-lateral membrane (IE), interspace basement membrane (ES), and basal-cell membrane (IS)

## Bond graph model

### Model description

The current BG-PCT encompasses cellular and lateral intercellular compartments, situated within the luminal and peritubular solutions. Figure 3 provides a schematic overview of the PCT epithelium, illustrating the arrangement of cellular and lateral intercellular space compartments along the tubule lumen. Five species, Na$^+$, K$^+$, Cl$^-$, HCO$_3^-$, and glucose, are considered within four distinct fluid compartments: the lumen (M), lateral interspace (E), cell interior (I), and basal solution (S). Five distinct membranes separate these compartments, each represented as a resistive module. These resistive modules correspond to the luminal cell membrane (MI), tight junction (ME), cell-lateral membrane (IE), interspace basement membrane (ES), and basal-cell membrane (IS). The order of the letters indicates the direction of positive flow. The BG-PCT model incorporates six different transporters, including co-transporters, exchangers, and an ATPase, which contribute to the diverse flux patterns observed within the system. A graphical overview of the complete BG-PCT structure is provided in Figs. 5 and 6.

### Model equations

This section details the equations governing the BG-PCT model, which are derived using the bond graph methodology by interconnecting resistive and capacitive modules. Within this model, a single bond can represent multiple coupled power transfer processes. Constant parameters are primarily drawn from Noroozbabaee et al. [9]. Leveraging the bond graph approach, we derive equations that describe both constituent transport across membranes (i.e., flows) and the development of compartmental potentials (i.e., efforts). Thus, these equations represent coupled sets of flows and efforts.

The epithelial transport system is conceptually divided into five resistive modules, representing the membrane structures (where dissipation occurs), and four capacitive modules, representing the solution compartments (where energy is stored). The system’s topology is illustrated diagrammatically in Fig. 5, showing how these modules are connected in series and parallel configurations via multiple ports.

Capacitive modules, characterized by half-cell absolute potentials, change in response to capacitive flows. These relationships are described by a set of ordinary differential equations derived from the capacitive modules ($\text {CM}_{\alpha }$). The capacitive flow variables define the rate of change of the potential variables. Specifically, at each capacitive module:

25 $$\begin{aligned} \text {CM}_{M}: \Sigma \nu&= 0 \rightarrow -f_{M} + J_{MI} + J_{ME} = 0, \end{aligned}$$

26 $$\begin{aligned} \text {CM}_{I}: \Sigma \nu&= 0 \rightarrow -f_{I} - J_{MI} + J_{IS} + J_{IE} = 0, \end{aligned}$$

27 $$\begin{aligned} \text {CM}_{S}: \Sigma \nu&= 0 \rightarrow -f_{S} - J_{IS} - J_{ES} = 0, \end{aligned}$$

28 $$\begin{aligned} \text {CM}_{E}: \Sigma \nu&= 0 \rightarrow -f_{E} + J_{ES} - J_{ME} - J_{IE} = 0. \end{aligned}$$

These equations express the conservation of flow at each capacitive junction. The rate of change of potential variables and the capacitive flows are represented as vector quantities. The capacitive relationships are expressed using a matrix $\textbf{B}$ (with components $B_{ij}$):

29 $$\begin{aligned} \frac{de_{i}}{dt} = - B_{ij}f_{j}. \end{aligned}$$

The driving forces across the resistive subsystems are determined by the differences in absolute potentials across the corresponding membranes. The transport equations, derived from the resistive modules ($\text {RM}_{\alpha \beta }$), relate the driving force variables to the corresponding flow variables (both vector quantities). For each resistive module:

30 $$\begin{aligned} \text {RM}_{MI}: \Sigma \mu&= 0 \rightarrow -e_{M} + X_{MI} + e_{I}= 0, \end{aligned}$$

31 $$\begin{aligned} \text {RM}_{IS}: \Sigma \mu&= 0 \rightarrow -e_{I} + e_{S} + X_{IS}= 0, \end{aligned}$$

32 $$\begin{aligned} \text {RM}_{ES}: \Sigma \mu&= 0 \rightarrow +e_{S} + X_{ES} - e_{E} = 0, \end{aligned}$$

33 $$\begin{aligned} \text {RM}_{ME}: \Sigma \mu&= 0 \rightarrow -e_{M} + e_{E} + X_{ME} = 0, \end{aligned}$$

34 $$\begin{aligned} \text {RM}_{IE}: \Sigma \mu&= 0 \rightarrow -e_{I} + e_{E} + X_{IE} = 0. \end{aligned}$$

These equations express the conservation of effort (or potential) across each resistive element. The conductance matrix $\textbf{L}$ (with components $L_{ij}$) defines the relationships between the resistive modules:

35 $$\begin{aligned} J_{i}= L_{ij} X_{j}. \end{aligned}$$

From the basic resistive module described in Section 2.2.1, the bond graph method yields the following relationships between flow and effort, describing constituent transport:

36 $$\begin{aligned} J^v_{\alpha \beta }&=L^v_{\alpha \beta }\big (\Delta p_{\alpha \beta }-\Delta \pi _{\alpha \beta }+\sum _{s}(1-\sigma ^{s}_{\alpha \beta }) \overline{c}^{s}_{\alpha \beta }\Delta \overline{\mu }^{s}_{\alpha \beta }\big ), \end{aligned}$$

37 $$\begin{aligned} J^{\text {Na}^+}_{\alpha \beta }&=\big (1-\sigma ^{\text {Na}^+}_{\alpha \beta }\big ) \overline{c}^{\text {Na}^+}_{\alpha \beta } J^v_{\alpha \beta }+\omega ^{\text {Na}^+}_{\alpha \beta }\overline{c}^{\text {Na}^+}_{\alpha \beta } \Delta \overline{\mu }^{\text {Na}^+}_{\alpha \beta }, \end{aligned}$$

38 $$\begin{aligned} J^{\text {K}^+}_{\alpha \beta }&=\big (1-\sigma ^{\text {K}^+}_{\alpha \beta }\big ) \overline{c}^{\text {K}^+}_{\alpha \beta } J^v_{\alpha \beta }+\omega ^{\text {K}^+}_{\alpha \beta } \overline{c}^{\text {K}^+}_{\alpha \beta } \Delta \overline{\mu }^{\text {K}^+}_{\alpha \beta },\end{aligned}$$

39 $$\begin{aligned} J^{\text {Cl}^-}_{\alpha \beta }&=\big (1-\sigma ^{\text {Cl}^-}_{\alpha \beta }\big ) \overline{c}^{\text {Cl}^-}_{\alpha \beta } J^v_{\alpha \beta }+\omega ^{\text {Cl}^-}_{\alpha \beta } \overline{c}^{\text {Cl}^-}_{\alpha \beta } \Delta \overline{\mu }^{\text {Cl}^-}_{\alpha \beta },\end{aligned}$$

40 $$\begin{aligned} J^{\text {HCO}_3^-}_{\alpha \beta }&= (1-\sigma ^{\text {HCO}_3^-}_{\alpha \beta } ) \overline{c}^{\text {HCO}_3^-}_{\alpha \beta } J^v_{\alpha \beta } + \omega ^{\text {HCO}_3^-}_{\alpha \beta } \overline{c}^{\text {HCO}_3^-}_{\alpha \beta }\Delta \overline{\mu }^{\text {HCO}_3^-}_{\alpha \beta },\end{aligned}$$

41 $$\begin{aligned} J^{Gluc}_{\alpha \beta }&= (1-\sigma ^{Gluc}_{\alpha \beta }) \overline{c}^{Gluc}_{\alpha \beta } J^v_{\alpha \beta } + \omega ^{Gluc}_{\alpha \beta } \overline{c}^{Gluc}_{\alpha \beta } \Delta \overline{\mu }^{Gluc}_{\alpha \beta }. \end{aligned}$$

These equations describe the coupled volumetric and solute fluxes across a membrane. To illustrate the incorporation of specific transporters, we consider the Na$^+$/K$^+$-ATPase pump and the K$^+$/Cl$^-$ co-transporter within the basal-cell (IS) membrane. The modified equations for this membrane are as follows:

42 $$\begin{aligned} J^v_{IS}&= L^v_{IS}(\Delta p_{IS} - \Delta \pi _{IS} + \sum _{s}(1 -\sigma ^{s}_{IS}) \overline{c}^{s}_{IS}\Delta \overline{\mu }^{s}_{IS})\end{aligned}$$

43 $$\begin{aligned} J^{\text {Na}^+}_{IS}&= (1-\sigma _{IS}^{\text {Na}^+}) \overline{c}^{\text {Na}^+}_{IS} J^v_{IS} + \omega ^{\text {Na}^+}_{IS} \overline{c}^{\text {Na}^+}_{IS}\Delta \overline{\mu }_{IS}^{\text {Na}^+} + J^{\text {NaK-Na}^+}_{IS},\end{aligned}$$

44 $$\begin{aligned} J^{\text {K}^+}_{IS}&= (1-\sigma _{IS}^{\text {K}^+}) \overline{c}^{\text {K}^+}_{IS} J^v_{IS} + \omega ^{\text {K}^+}_{IS} \overline{c}^{\text {K}^+}_{IS}\Delta \overline{\mu }_{IS}^{\text {K}^+}+J^{\text {KCl-K}^+}_{IS},\end{aligned}$$

45 $$\begin{aligned} J^{\text {Cl}^-}_{IS}&= (1-\sigma _{IS}^{\text {Cl}^-}) \overline{c}^{\text {Cl}^-}_{IS} J^v_{IS} + \omega ^{\text {Cl}^-}_{IS} \overline{c}^{\text {Cl}^-}_{IS}\Delta \overline{\mu }_{IS}^{\text {Cl}^-}+J^{\text {KCl-Cl}^{-}}_{IS},\end{aligned}$$

46 $$\begin{aligned} J^{\text {HCO}_3^{-}}_{IS}&= (1-\sigma _{IS}^{\text {HCO}_3^{-}} ) \overline{c}^{\text {HCO}_3^{-}}_{IS} J^v_{IS} + \omega ^{\text {HCO}_3^{-}}_{IS} \overline{c}^{\text {HCO}_3^-}_{IS} \Delta \overline{\mu }_{IS}^{\text {HCO}_3^-},\end{aligned}$$

47 $$\begin{aligned} J^{\text {Gluc}}_{IS}&= (1-\sigma ^{\text {Gluc}}_{IS}) \overline{c}^{\text {Gluc}}_{IS} J^v_{IS} + \omega ^{\text {Gluc}}_{IS} \overline{c}^{\text {Gluc}}_{IS}\Delta \overline{\mu }_{IS}^{\text {Gluc}}. \end{aligned}$$

The terms $J^{\text {NaK-Na}^{+}}_{IS}$, $J^{\text {KCl-K}^{+}}_{IS}$, and $J^{\text {KCl-Cl}^{-}}_{IS}$ represent the fluxes due to the Na$^+$/K$^+$-ATPase pump and the K$^+$/Cl$^-$ co-transporter, respectively. Further details regarding these transporters are provided in Equations S1, and S5, S7 (Online Resource 1).

The following definitions are also relevant:

48 $$\begin{aligned} -\frac{dV_\alpha }{dt}&= - f^v_{\alpha }, \end{aligned}$$

49 $$\begin{aligned} \frac{dn^{s}_{\alpha }}{dt}&= - f^{s}_{\alpha }\quad \text {where}\quad n^{s}_{\alpha } = c^{s}_{\alpha } V_\alpha ,\end{aligned}$$

50 $$\begin{aligned} \Delta \mu ^{s}_{\alpha \beta }&= \frac{\Delta \pi ^{s}_{\alpha \beta }}{ \overline{c}^{s}_{\alpha \beta }}. \end{aligned}$$

The rate of free-energy change of the half-cell-type capacitive module is expressed as follows, using the vector expression of flow *f* and effort *e*

51 $$\begin{aligned} -\frac{d G}{dt} = f_ie_i. \end{aligned}$$

with *G* being the free-energy, and implicit summation assumed for the repeated index.

### Model implementation

In this work, the BG-PCT model is bathed on both the luminal and peritubular sides by solutions of equal concentration. Baseline bath and lumen conditions are those reported in Table S3, and the choices for the model parameters are disclosed in Table S2 (Online Resource 1). The PCT Python code is available for download on GitHub: https://github.com/iNephron/BondGraph_PCT.

### Numerical simulations

A series of numerical experiments was conducted to evaluate the capacity of the BG-PCT to represent the physiological transport processes of the kidney. Steady-state solutions were used as the basis for analysis.

The evaluation proceeded in three stages. First, the robustness of the model was assessed by examining the sensitivity of steady-state solutions to variations in initial conditions and time steps. Second, in silico inhibition studies were performed, targeting key transporters with a more detailed secondary analysis of peritubular Na$^+$/K$^+$-ATPase. Finally, the impact of luminal Na$^+$ and Cl$^-$ concentration on PCT transport was investigated. Na$^+$ and Cl$^-$ concentrations were increased in parallel to maintain electroneutrality.

For each of these simulations, we used the odeint function from the scipy.integrate module to solve the differential equations [26]. A maximum time step of $\Delta t = 0.1~\text {s}$ was selected to ensure numerical stability and accuracy of the solutions; larger time steps may reduce accuracy and compromise convergence. Unless otherwise stated, simulations were run until a steady state was achieved (typically within 200 min of simulated time).

## Results

This study describes the BG-PCT, a thermodynamically consistent representation of fluid and solute transport in the PCT that was achieved through combining a series of parameterized resistive and capacitive modules. The numerical simulations, detailed in the following sections, indicate that the model predictions align with expected physiological behavior.

### Structural analysis

**Fig. 7:**
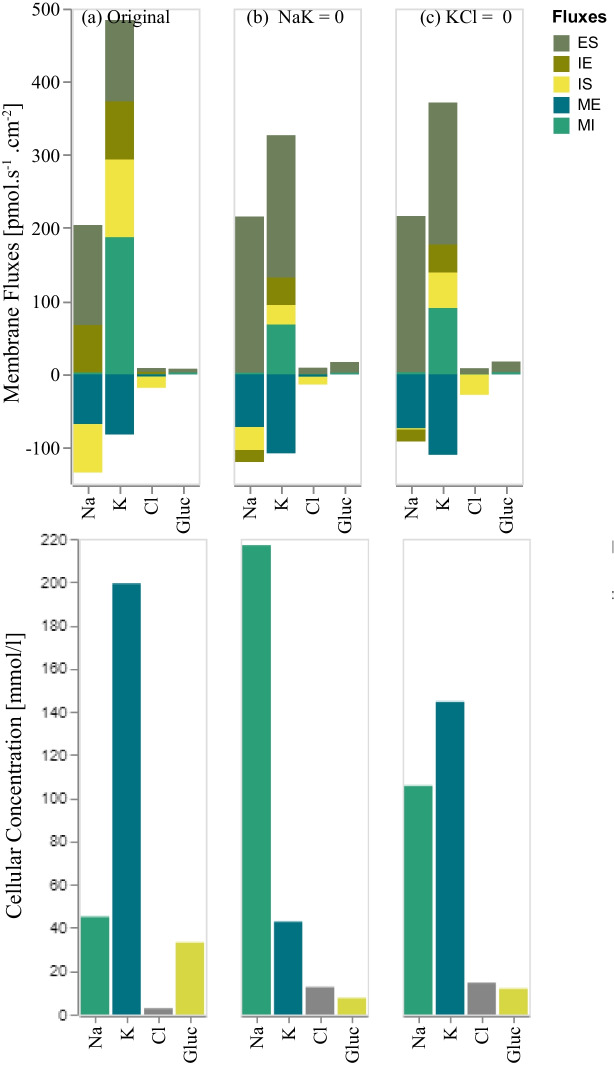
Changes in the membrane fluxes and cellular concentrations due to the inhibition of peritubular transporters. Top: Total membrane fluxes for Na$^+$, K$^+$, Cl$^-$, and glucose across five stacked membranes (IS, ME, MI, IE, and ES). **a** Original full model (control scenario). **b** Na$^+$/K$^+$-ATPase inhibition. **c** K$^+$/Cl$^-$ co-transporter inhibition. Bottom: Corresponding steady-state cellular solute concentrations

**Fig. 8:**
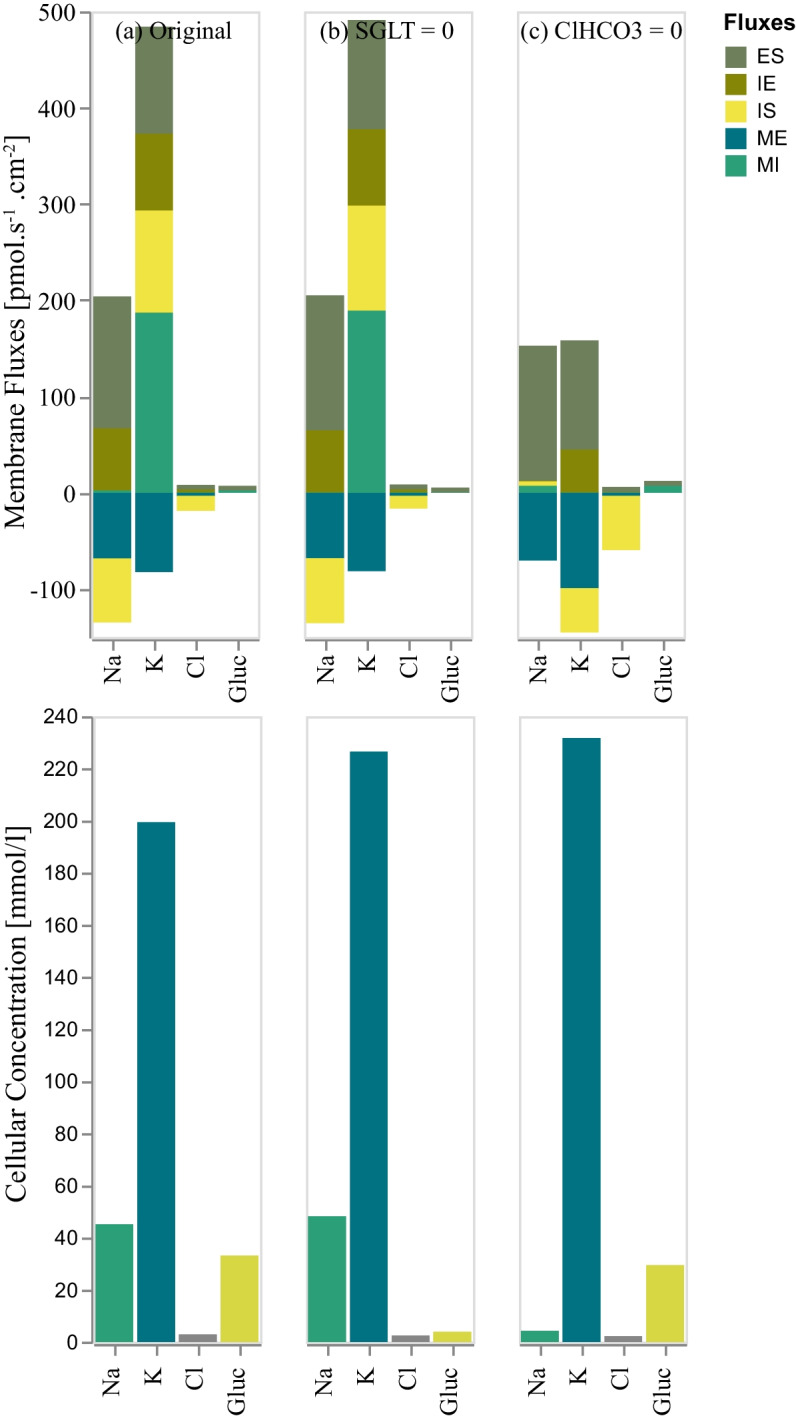
Changes in the membrane fluxes and cellular concentrations due to the inhibition of transporters on the apical cell membrane. Top: Total membrane fluxes for Na$^+$, K$^+$, Cl$^-$, and glucose across five stacked membranes (IS, ME, MI, IE, and ES). **a** Original full model (control scenario). **b** SGLT inhibition. **c** Cl$^-$/HCO$_3^-$ exchanger inhibition. Bottom: Corresponding steady-state cellular solute concentrations

We performed a structural analysis to explore the response of the BG-PCT model to various physiological disturbances. This analysis involved selectively inhibiting (1) peritubular and (2) apical transporters and quantifying the resulting changes in net fluxes and steady-state concentrations. Inhibition was performed by setting the coupling transport coefficient of the respective transporters to zero. In each case of selective inhibition, the unperturbed BG-PCT model served as the control configuration, against which transporter-specific perturbations were compared (Figs. 7a and 8a).

#### Inhibition of peritubular transporters

To explore the contribution of the peritubular transporters, we separately inhibited the Na$^+$/K$^+$-ATPase and the K$^+$/Cl$^-$ co-transporter on the cell-basal (IS) and cell-lateral (IE) membranes. Inhibiting Na$^+$/K$^+$-ATPase decreased cell-basal Na$^+$ efflux and halted K$^+$ influx. This results in a marked increase in intracellular Na$^+$ concentration (from 45 to 217 mmol/L) and a decrease in K$^+$ concentration (from 199 to 42 mmol/L). Due to the cross-solute coupling, these disruptions also resulted in a decrease in intracellular glucose and an increase in Cl$^-$ concentration (Fig. 7b). The accumulation of Cl$^-$ is likely due to a diminished capacity for efflux through the K$^+$/Cl$^-$ co-transporter, which is reflected in the reduced IS flux. The mechanism for reduced steady-state glucose concentration is less obvious, but appears to be the result of a decrease in apical availability due to greater flux via the lateral interspace (ES).

Removing the K$^+$/Cl$^-$ co-transporter reduced intracellular K$^+$ and glucose concentrations while it increased Na$^+$ and Cl$^-$ concentrations (Fig. 7c). Similar to the inhibition of Na$^+$/K$^+$-ATPase, the decrease of steady-state intracellular glucose concentration appears to have arisen as a byproduct of increased paracellular (ES) glucose flux. The reduced steady-state Na$^+$ concentration also appears to arise from increased flux via the paracellular pathway, combined with an elimination of the cell-basal (IS) Na$^+$ influx.

#### Inhibition of apical membrane transporters

Blocking apical SGLT (sodium–glucose cotransporter) resulted in a significant reduction in glucose concentration from 33.16 mmol/L to 3.8 mmol/L. However, the inhibition did not cause notable changes in epithelial Na$^+$ or Cl$^-$ fluxes, demonstrating that SGLT primarily impacts glucose and, to a lesser extent, K$^+$ fluxes (Fig. 8b).

The inhibition of the Cl$^-$/HCO$_3^-$ exchanger led to pronounced changes in steady-state solute concentrations. Cl$^-$ concentration decreased from 3.0 to 2.1 mmol/L, while Na$^+$ concentration dropped significantly from 45.0 to 4.2 mmol/L. Conversely, K$^+$ concentration increased from 199.3 to 231.4 mmol/L (Fig. 8c). By eliminating the apical Cl$^-$ influx, the operation mode of the Cl$^-$/K$^+$ co-transporter appears to reverse, contributing to increased basal (IS) K$^+$ and Cl$^-$ influx.

### Salt sensitivity

**Fig. 9:**
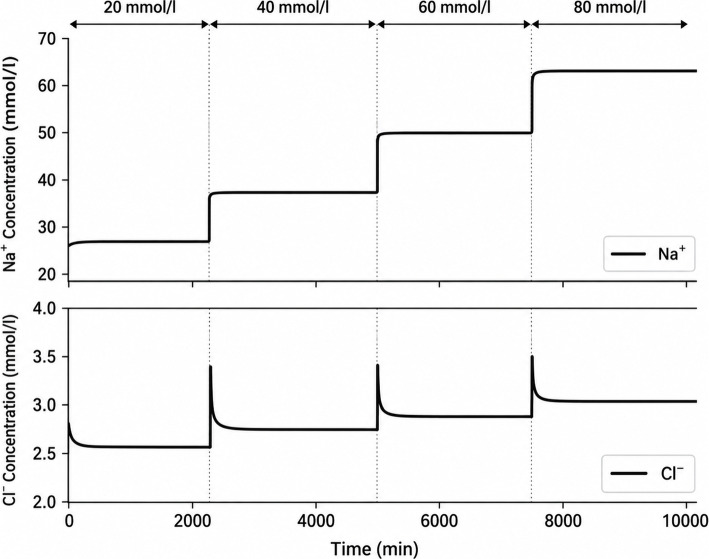
Effect of changes in luminal and peritubular Na$^+$ and Cl$^-$ concentrations on the cellular system. Both peritubular and luminal Na$^+$ and Cl$^-$ concentrations are increased in a step-wise manner; the values at the top of the figure indicate peritubular and luminal concentrations of Na$^+$ and Cl$^-$

When raising the peritubular and luminal Na$^+$ and Cl$^-$ concentrations in 20 mmol/L increments, we observed concomitant step-wise increases in cellular Na$^+$ and Cl$^-$ concentration (Fig. 9). The time course of cellular ion concentration dynamics following a step-wise increase in peritubular and luminal concentration was markedly different between ions. Cellular Cl$^-$ exhibited a sharp, brief overshoot that rapidly returned to an increasing baseline, whereas Na$^+$ exhibited a smoother transition between concentration states. In addition, the relationship between extracellular and intracellular concentration was species-dependent. Steady-state cellular Na$^+$ concentration increased by approximately the same amount with each step increase in environmental concentration, whereas the step-wise increase in concentration of cellular Cl$^-$ diminished progressively.

## Discussion

This study introduces the BG-PCT model, a novel application of bond graph modeling that extends existing physiological bond graph frameworks for solute transport by introducing consideration of volumetric flux. While less physiologically comprehensive than existing kinetic models of the PCT (e.g., [9]), the BG-PCT successfully reproduces experimental physiology results within a modeling paradigm that enforces thermodynamic consistency. Bond graphs facilitate modularity and extensibility, providing a robust foundation for future model development. To support reusability, the BG-PCT model and associated tools are freely available on GitHub under an open-source license (https://github.com/iNephron/BondGraph_PCT), providing a valuable resource for researchers, especially those new to the field, who wish to investigate complex physiological phenomena using epithelial transport models.

The predictions of the BG-PCT model in response to physiological perturbations broadly aligned with experimental observations. For example, the dynamic coordination between basolateral Na$^+$/K$^+$-ATPase and apical Na$^+$ transport predicted by our model is a well-established mechanism that plays a crucial role in regulating transepithelial Na$^+$ flux [27–30]. The impact of transporter inhibition on the remaining solutes considered by the BG-PCT was also consistent with observations from recent clinical studies [31, 32]. While our exploration of the influence of luminal salt concentration did not duplicate the experiment depicted in [33, Figure 8], which focused on urinary bladder epithelial cells rather than kidney epithelium, a qualitative comparison between these models reveals similar system responses. These results suggest that, even in its minimal form, the transport dynamics of the BG-PCT model remains faithful to the underlying physiology.

Beyond reproducing expected physiological behavior, the structural inhibition analyses provide mechanistic insight into how specific transporters regulate key renal functions. For instance, the pronounced reduction in intracellular glucose concentration following SGLT inhibition reflects the central role of apical sodium–glucose cotransport in proximal tubular glucose reabsorption and is consistent with the therapeutic mechanism of SGLT inhibitors used in diabetes management [34]. Similarly, perturbations of the basolateral Na$^+$/K$^+$-ATPase markedly altered intracellular Na$^+$ and K$^+$ levels, highlighting the dependence of transepithelial Na$^+$ reabsorption and fluid balance on this pump, processes directly linked to blood pressure regulation and chronic kidney disease progression. These examples illustrate how the BG-PCT framework can serve not only as a mechanistic physiology model but also as an in silico testbed to explore the functional consequences of transporter dysfunction, pharmacological inhibition, or disease-related parameter changes. Although the present study does not aim to provide quantitative clinical predictions, the model establishes a structured platform for future investigations of transporter-targeted therapies, biomarker development, and pathophysiological hypotheses in renal epithelial transport.

Bond graph methods have recently been gaining popularity for creating reusable and modular models of physiological systems, ensuring physical plausibility and thermodynamic consistency (see, for example, [15–17, 20, 35–40]). By representing energy transfer across different physical domains (e.g., coupled biochemical reactions and mechanical contraction, or ion movement and electrical potential changes), these models are well-suited to capture the complex interplay of processes within physiological systems and ensure thermodynamically consistent model compositions.

Despite the clear advantages of bond graph modeling, its application to epithelial cell models has been limited. Existing bond graph models often neglect volumetric fluxes, a critical factor in understanding solute and water transport [15]. While the pioneering work of Imai et al. on network thermodynamics addressed volumetric fluxes in epithelial cell models [21, 22, 41], the associated publications lack the level of detail required for reproducibility and reusability, thus hindering comprehensive system analysis [42–45].

To address these limitations, we present the BG-PCT model: a well-tested and FAIR-compliant bond graph model of epithelial transport in the PCT. Its modular design is aided by the underlying thermodynamic consistency and facilitates future extensibility and reusability. By demonstrating the ability of the BG-PCT model to reproduce previous findings and showcasing its flexibility through structural analysis, we establish a robust foundation for future investigations of complex physiological phenomena. By adjusting the location, density, or types of transporters present, the model can be adapted to capture species, organ, or regional variations. We have provided tools in the BG-PCT GitHub repository to facilitate users in creating configurations specific to their needs.

Although capable of reproducing some physiological epithelial transport dynamics, the BG-PCT model, in its current minimal form, has limitations that affect its physiological accuracy and predictive power. The absence of key transporters, such as the Na$^+$/H$^+$ exchanger, can lead to unrealistic ion concentrations and electrical potentials, thus limiting the model’s representation of PCT function [46]. Similarly, the simplified representation of buffer systems restricts the ability of the model to accurately simulate acid-base balance, a crucial aspect of renal physiology [47].

Water transport in the proximal tubule is known to occur predominantly through aquaporins, particularly AQP1 and AQP7, which confer high membrane water permeability [48]. In the present BG-PCT model, water movement is represented phenomenologically through the hydraulic permeability parameter $L^v$, which effectively captures the aggregate permeability of both the lipid bilayer and aquaporin-mediated pathways. Consequently, aquaporins are not modeled explicitly as channel proteins but are implicitly incorporated within this lumped permeability term. While this minimal representation is sufficient for reproducing steady-state osmotic–hydrostatic coupling, the modular bond graph architecture readily allows incorporation of explicit AQP elements and dynamic regulation of $P_f$ in future extensions.

Similarly, the Na$^+$/K$^+$-ATPase is currently modeled using fixed transport coefficients, representing a near-equilibrium approximation of pump activity. Future work will leverage the thermodynamic scalability of the bond graph framework to replace these constants with non-linear constitutive relations [20]. This would allow the model to dynamically account for local ATP/ADP ratios and hormonal modulation by allowing these coefficients to vary dynamically within the same bond graph framework.

Future research should prioritize reintroducing additional transporters previously implemented in earlier kinetic PCT models, such as the Na$^+$/H$^+$ exchanger (NHE3), and a more detailed representation of intracellular buffer systems to enhance its predictive capabilities. Extending the model to include other epithelial segments, such as the distal tubule and collecting duct, would further broaden its applicability to renal physiology research. Addressing these limitations will unlock the potential of the BG-PCT model for investigating a wider range of physiological processes within the renal system and beyond. While the present study focuses on steady-state solutions and transient responses to specific perturbations, a more comprehensive dynamical systems analysis could provide additional insight into the model behavior. In particular, bifurcation analysis of key transport parameters may help identify transitions between physiological and pathological regimes. Similar approaches have been applied previously in the analysis of neural population models to investigate stability and regime transitions in biological systems [49].

## Conclusions

This study contributes to the development of a standardized repository of reusable, FAIR-compliant transporter modules. To ensure the reliability and validity of composite models, we developed the BG-PCT model by assembling individual transport process models within a thermodynamically consistent bond graph framework. This consistency is crucial for the arbitrary composition of models, a necessary step towards realizing a comprehensive virtual nephron: the iNephron.

As we move towards the arbitrary composition of models required for a virtual nephron (or any epithelial transport system), thermodynamic consistency emerges as a fundamental requirement. The bond graph method, which we employed to define both individual process models and their composition into a comprehensive model of epithelial cellular transport, provides a robust approach that inherently incorporates thermodynamics and ensures consistency across the multiple interacting physical domains.

### Supplementary information

**ESM 1:** Model equations and simulation parameters for the bond graph model of the proximal convoluted tubule (BG-PCT)

## Supplementary Information

Below is the link to the electronic supplementary material.

Supplementary file 1 (pdf 260 KB)

## Data Availability

No datasets were generated or analysed during the current study.
