# Supplementary material for: A thermodynamically consistent approach to modeling epithelial solute and water transport in the proximal convoluted tubule
Source: J Biol Phys. 2026 Jun 19;52(1):22. doi: 10.1007/s10867-026-09713-0 (PMC13282429; doi:10.1007/s10867-026-09713-0)
Supplement: Supplementary file 1 — (pdf 260 KB) [file 10867_2026_9713_MOESM1_ESM.pdf]

Supplementary Material

A thermodynamically consistent approach to modelling epithelial solute and water transport in the proximal convoluted tubule

Leyla Noroozbabaee*, Jarrah M. Dowrick, Pablo J. Blanco, David P. Nickerson

Journal of Biological Physics

***Corresponding author:** MERLN Institute for Technology-Inspired Regenerative Medicine, Maastricht University, Maastricht, The Netherlands; leyla.noroozbabaee@maastrichtuniversity.nl.

In this supplementary material, we include further details on the BG-PCT model membrane fluxes and present information on the full simulation experiments used to produce the results presented in the main manuscript.

Transporter fluxes: Coupled solute fluxes

Coupled solute fluxes in this model fall into three categories: *simple co-transporters*, *simple exchangers*, and *complex exchangers*. These transporters are described using linear non-equilibrium thermodynamics, where solute permeation rates are proportional to the electrochemical driving force of the aggregate species, with a single permeation coefficient.

Simple co-transporters

Examples include the peritubular K^+/Cl^- co-transporter and the luminal Na^+/Glucose co-transporter, where the fluxes of the two species across the co-transporter follow a 1:1 stoichiometry. The fluxes through these transporters are defined as:

$$\begin{bmatrix} J_{IS}^{K^+} \\ J_{IS}^{Cl^-} \end{bmatrix} = L_{(K^+,\, Cl^-)} \begin{bmatrix} 1 & 1 \\ 1 & 1 \end{bmatrix} \begin{bmatrix} \bar{\mu}_{IS}^{K^+} \\ \bar{\mu}_{IS}^{Cl^-} \end{bmatrix}, \tag{S1}$$

$$\begin{bmatrix} J_{MI}^{Na^+} \\ J_{MI}^{Gluc} \end{bmatrix} = L_{(Na^+,\, Gluc)} \begin{bmatrix} 1 & 1 \\ 1 & 1 \end{bmatrix} \begin{bmatrix} \bar{\mu}_{MI}^{Na^+} \\ \bar{\mu}_{MI}^{Gluc} \end{bmatrix}. \tag{S2}$$

Simple exchangers

An example is the Cl^-/HCO_3^- exchanger, defined as:

$$\begin{bmatrix} J_{MI}^{Cl^-} \\ J_{MI}^{HCO_3^-} \end{bmatrix} = L_{(Cl^-,\, HCO_3^-)} \begin{bmatrix} 1 & -1 \\ -1 & 1 \end{bmatrix} \begin{bmatrix} \bar{\mu}_{MI}^{Cl^-} \\ \bar{\mu}_{MI}^{HCO_3^-} \end{bmatrix}. \tag{S3}$$

Complex exchangers

Examples include the peritubular Na^+/HCO_3^- and $Na^+/2HCO_3^-/Cl^-$ exchangers, represented as:

$$\begin{bmatrix} J_{IS}^{\mathrm{Na}^+} \\ J_{IS}^{\mathrm{HCO}_3^-} \end{bmatrix} = L_{(\mathrm{Na}^+,\,\mathrm{HCO}_3^-)} \begin{bmatrix} 1 & 3 \\ 3 & 9 \end{bmatrix} \begin{bmatrix} \bar{\mu}_{IS}^{\mathrm{Na}^+} \\ \bar{\mu}_{IS}^{\mathrm{HCO}_3^-} \end{bmatrix}. \tag{S4}$$

Transporter fluxes: Active solute fluxes

The model includes one ATPase: the peritubular Na^+/K^+-ATPase, which exchanges three cytosolic Na^+ ions for two peritubular K^+ ions. The fluxes due to Na^+/K^+-ATPase activity are:

$$J_{\alpha\beta}^{\mathrm{NaK}} = L_{(\mathrm{NaK})}\left(D_{\alpha\beta}^{\mathrm{ATP}} - 3\,\Delta\bar{\mu}_{\alpha\beta}^{\mathrm{Na}^+} + 2\,\Delta\bar{\mu}_{\alpha\beta}^{\mathrm{K}^+}\right), \tag{S5}$$

$$J_{\alpha\beta}^{\mathrm{NaK\text{-}Na}^+} = 3\,J_{\alpha\beta}^{\mathrm{NaK}}, \tag{S6}$$

$$J_{\alpha\beta}^{\mathrm{NaK\text{-}K}^+} = -2\,J_{\alpha\beta}^{\mathrm{NaK}}. \tag{S7}$$

Simulation parameters

A collection of tables detailing the initial conditions, boundary conditions, and parameter values used in all simulations.

Table S1: Global constants used in the bond graph model of the proximal convoluted tubule (BG-PCT).

Constant	Definition	Value	Unit
R	Universal gas constant	8.314	$\mathrm{J\,mol^{-1}\,K^{-1}}$
T	Temperature	273.15	K
F	Faraday's constant	96.5×10^3	$\mathrm{C\,mol^{-1}}$
z^{Na^+}	Na^+ valency	+1	—
z^{K^+}	K^+ valency	+1	—
z^{Cl^-}	Cl^- valency	−1	—
$z^{\mathrm{HCO}_3^-}$	HCO_3^- valency	−1	—
z^{Gluc}	Glucose valency	0	—

Table S2: Membrane property parameter values and initial conditions. ME = mucosal–epithelial, MI = mucosal–interstitial, IE = interstitial–epithelial, ES = epithelial–serosal, IS = interstitial–serosal.

**Parameter**	**ME**	**MI**	**IE**	**ES**	**IS**
		Membrane area [cm^2 / $cm^2_{epithelial}$]			
$A_{\alpha\beta}$	0.001	36.0	36.0	2.0×10^{-2}	1.0
		Hydraulic permeability $\times RT$ [$cm\,s^{-1}\,Osmol^{-1}$]			
$L^v_{\alpha\beta}$	4.0×10^{0}	2.0×10^{-4}	2.0×10^{-4}	6.0×10^{0}	2.0×10^{-4}
		Permeability coefficient $\omega^s_{\alpha\beta}$ [$cm\,s^{-1}$]			
Na^+	2.6×10^{-2}	0	3.9×10^{-9}	5.0×10^{-2}	1.0
K^+	2.9×10^{-1}	2.5×10^{-7}	2.0×10^{-6}	7.0×10^{-2}	2.0×10^{-6}
Cl^-	1.0×10^{0}	0	0	6.0×10^{-2}	0
HCO_3^-	8.0×10^{-2}	1.0×10^{-8}	0	5.0×10^{-2}	0
Glucose	1.6×10^{-2}	0	7.5×10^{-6}	3.0×10^{-2}	7.5×10^{-6}
		Reflection coefficient $\sigma^s_{\alpha\beta}$			
Na^+	0.75	1.0	1.0	0.0	1.0
K^+	0.60	1.0	1.0	0.0	1.0
Cl^-	0.30	1.0	1.0	0.0	1.0
HCO_3^-	0.90	1.0	1.0	0.0	1.0
Glucose	1.0	1.0	1.0	0.0	1.0
		Coupled transport pathways [$mol^2\,J^{-1}\,s^{-1}\,cm^{-2}$]			
K^+–Cl^-	—	—	6.5×10^{-5}	—	5.0×10^{-7}
Na^+–2 HCO_3^-/Cl^-	—	—	1.0×10^{-7}	—	5.0×10^{-7}
Na^+–3 HCO_3^-	—	—	5.0×10^{-11}	—	5.0×10^{-10}
Na^+–Glucose	—	5.0×10^{-11}	—	—	—
Cl^-/HCO_3^-	—	2.0×10^{-9}	—	—	—
		Active transport [$mol^2\,J^{-1}\,s^{-1}\,cm^{-2}$]			
Na^+/K^+-ATPase	—	—	6.0×10^{-11}	—	1.0×10^{-9}

Table S3: Compartment property parameter values and initial conditions. M = mucosal, E = epithelial, I = interstitial, S = serosal.

**Parameter**	**M**	**E**	**I**	**S**
		Concentration c_α^s [mol/L]		
Na^+	0.14	0.14	0.02	0.14
K^+	4.9×10^{-3}	4.6×10^{-3}	0.10	4.9×10^{-3}
Cl^-	1.7×10^{-2}	0.11	1.6×10^{-2}	0.113
HCO_3^-	0.024	2.0×10^{-2}	1.5×10^{-3}	0.024
Glucose	0.005	7.7×10^{-3}	2.4×10^{-2}	0.005
		Electrical potential [mV]		
ψ_α	Eq. 20	Variable	Variable	0.0
		Hydrostatic pressure [mmHg]		
p_α^0	15.0	−0.1	15.0	9.0
